# The impact of bacteremia on lipoprotein concentrations and patient’s outcome: a retrospective analysis

**DOI:** 10.1007/s10096-019-03543-w

**Published:** 2019-04-13

**Authors:** Alex Pizzini, Katharina Kurz, Dorothea Orth-Hoeller, Manfred Fille, Jasmin Rabensteiner, Fabian Lunger, Lukas Lunger, Christoph Tschurtschenthaler, Ivan Tancevski, Robert Krause, Cornelia Lass-Floerl, Günter Weiss, Rosa Bellmann-Weiler

**Affiliations:** 1 Department of Internal Medicine II, Infectious Diseases, Pneumology, Rheumatology, Medical University of Innsbruck, Anichstraße 35, Innsbruck, Austria; 2 Department of Microbiology and Hygiene, Medical University of Innsbruck, Schöpfstraße 41, Innsbruck, Austria; 3 Section of Infectious Diseases and Tropical Medicine, Medical University of Graz, Auenbruggerplatz 15, Graz, Austria; 4 Department of Gynecologic Endocrinology and Reproductive Medicine, Medical University of Innsbruck, Anichstraße 35, Innsbruck, Austria; 5 Department of Internal Medicine I, Gastroenterology, Hepatology, Endocrinology and Metabolism, Medical University of Innsbruck, Anichstraße 35, Innsbruck, Austria

**Keywords:** Lipoprotein, HDL, LDL, Triglycerides, Bacteremia, Gram species

## Abstract

Bacteremia is a major clinical challenge requiring early treatment. Metabolic alterations occur during bacteremia, and accordingly plasma concentrations of lipoproteins LDL-C and HDL-C are substantially changed. We questioned whether bacteremia with Gram-negative versus Gram-positive bacteria causes contrasting changes of lipoprotein levels in order to differentiate between the 2-g stain types and if there is a relation with outcome parameters namely ICU-admission, 30-day mortality, duration of hospitalization. This is a retrospective dual-center cross-sectional study, including 258 patients with bacteremia. Plasma lipid levels were analyzed within 48 h to positive blood culture. Upon admission, HDL-C, LDL-C, and total cholesterol (*p* = 0.99) in plasma did not significantly differ between patients with Gram-negative and Gram-positive bacteremia, while significantly higher triglyceride concentrations were found in Gram-negative bacteremia (*p* < 0.05). 30-day mortality and ICU admission were associated with lower LDL-C and HDL-C concentrations as compared to survivors and non-ICU patients, and patients with HDL-C < 20 mg dl^−1^ and LDL-C < 55 mg dl^−1^ had a relative risk (RR) of 2.85 for ICU therapy requirement and RR = 2 of death within 30 days. Reduced HDL-C and LDL-C concentrations were associated with adverse patient’s outcome in bacteremia. Discrimination between Gram-negative and Gram-positive pathogens upon lipoprotein patterns is unlikely.

## Introduction

Bacteremia occurs in 140–160 individuals per 100,000 per year in high-income countries and is associated with significant morbidity and mortality even years after the infection [1, 2]. Bacteremia accompanied by clinical symptoms may cause a dysregulated multi-system inflammatory response encompassing physiologic, biologic and biochemical abnormalities, denoted as sepsis [3].

Blood cultures (BC) are the gold standard for the diagnosis of bacteremia but are only found positive in one third of sepsis cases, and in many septic patients, no causative agent can be identified [4]. Yet, to ensure therapeutic success and to reduce intra-hospital mortality, prompt identification of septic patients and immediate empiric anti-microbial therapy are pivotal [5]. However, BC results are typically available not prior to 48–96 h, limiting the initiation of a targeted antibiotic regimen, a fact that is often compensated through liberal use of broad-spectrum antibiotics based on specific risk factors of patients, site of infection, and on local occurrence of anti-microbial resistance [5, 6].

Therefore, focus has been put on the identification of additional markers and/or tests to enhance initial microbiologic evaluation. High-density lipoprotein cholesterol (HDL-C) appears to have evolved as part of the innate immune system playing an essential role in the acute-phase (AP) response and sepsis [7]. Inflammatory diseases lead to marked changes in HDL-C metabolism, decreasing plasma HDL-C levels and altering structure and conformation of HDL-C particles [7]. To date, the observed decrease in HDL-C plasma levels during the AP response remains incompletely understood, and a variety of different mechanisms have been suggested [8, 9]. It has been shown in vitro and in vivo that HDL-C can bind and neutralize lipopolysaccharide (LPS) from Gram-negative bacteria, yielding a clue to the importance of HDL-C during the AP [10, 11]. Of note, metabolic reprogramming involving subtle alterations of multiple steps of lipid homeostasis and energy metabolism occurs in subjects with sepsis and has been shown to impact on the outcome of the disease [12].

We, therefore, aimed to evaluate the effect of manifest bacteremia on plasma cholesterol, particularly HDL-C and low-density lipoprotein cholesterol (LDL-C) concentrations and to investigate to what extent alterations of the lipid profile at the time of a positive BC may indicate bacteremia with either Gram-positive or Gram-negative bacteria. A further goal was to analyze the correlation between lipoprotein concentrations and prognostic parameters, namely the length of hospital stay, 30-day mortality, and ICU admission.

## Methods

### Subjects

We retrospectively identified 221 eligible patients with community-acquired bacteremia seen between January 2005 and December 2013 at the Department of Hygiene and Medical Microbiology of the Innsbruck University Hospital. We additionally enrolled 37 patients from the University Hospital of Graz, meeting the same inclusion/exclusion criteria.

Patients between 18 and 90 years of age with a positive BC were included. Study inclusion also required the presence of plasma cholesterol and lipid levels, determined within the first 48 h to positive BC.

Exclusion criteria were pregnancy, total parenteral nutrition, malignancy, and more than one bacterial strain in the BC.

Following available data were collected: blood cell counts, C-reactive protein (CRP), procalcitonin (PCT), triglycerides (TG), HDL-C, LDL-C, total cholesterol, creatinine, body mass index (BMI), presence of diabetes, statin therapy, blood-culture results, duration of hospitalization, ICU treatment, and mortality < 30 days.

### Ethics approval and consent to participate

All procedures performed in the present study involving human participants were in accordance with the ethical standards of the Institutional and/or National Research Committee and with the 1964 Helsinki declaration and its later amendments and were performed after approval of the Ethics Committee of the Medical University of Innsbruck (Nr. UN4118, Session 292/4.29).

### Statistical analyses

Data distribution was estimated via histogram and Kolmogorov-Smirnov test in order to assess the fit of data to a normal distribution. For normally distributed data, baseline characteristics of the two groups were compared using *t* test, chi-square, and Fisher’s exact test, where appropriate. For non-parametric data, the Mann-Whitney *U* test was applied. Bivariate relations between *X* and *Y* were analyzed with the Spearman correlation coefficient. A linear regression analysis model was generated to characterize relationships between dependent and independent variables. Statistical analyses were performed with the SPSS 24.0 (IBM Corp., Armonk, NY, USA). A two-sided *p* value of < 0.05 was considered significant.

## Results

Two hundred fifty-eight patients met our inclusion criteria and were enrolled in the analysis. The baseline characteristics of our cohort are shown in Table 1. One hundred thirty-seven patients (53.1%) had bacteremia with Gram-negative and 121 patients (46.9%) with Gram-positive bacteria. The most frequently diagnosed bacteria were *E. coli* species (*n* = 108, 41.7%), followed by *Coagulase-negative staphylococci* (*n* = 48, 18.6%) and *Staphylococcus aureus* (*n* = 32, 12.4%). The detailed list of all detected bacteria is shown in Table 2. While subjects with Gram-positive bacteremia were significantly younger (*p* < 0.01), Gram-negative bacteremia was significantly more prevalent in women (*p* < 0.01). BMI did not significantly differ between the two groups (*p* = 0.06), and no difference in diabetes prevalence (*p* = 0.89) or frequency of statin therapy (*p* = 0.19) was found (Table 1).

**Table 1:** Baseline characteristics. Mean ± standard deviation. *BMI* body mass index, *CRP* C-reactive protein, *PCT* procalcitonin, *HDL-C* high-density lipoprotein cholesterol, *LDL-C* low-density lipoprotein cholesterol, *TG* triglycerides, *WBC* white blood cells

Parameter	*n*	Gram stain		
		Negative	Positive	*p*
No. of patients^b^	258	137 (53.1)	121 (46.9)	
Age (years)^a^	258	76 (21)	66 (28)	< 0.01**
BMI (kg m^−2^)	144	25.1 ± 4.7	26.6 ± 4.9	0.06
Gender male	136	59 (43.4)	77 (56.6)	< 0.01**
Gender female	122	78 (63.9)	44 (36.1)	< 0.01**
Influencing factors^b^				
Diabetes	63	34 (54)	29 (46)	0.89
Statin therapy	58	28 (48.3)	30 (51.7)	0.19
Laboratory parameters				
CRP (mg dl^−1^)^a^	258	15.92 (14.83)	11.61 (15.83)	< 0.01**
PCT (μg l^−1^)^a^	109	5.31 (11.82)	1.44 (6.24)	0.01*
HDL-C (mg dl^−1^)^a^	258	27 (27)	32 (28)	0.11
LDL-C (mg dl^−1^)^a^	258	72 (45)	74 (55)	0.30
TG (mg dl^−1^)^a^	258	138 (99)	115 (79)	< 0.05*
Total cholesterol (mg dl^−1^)^a^	258	139 (40)	135 (69)	0.99
Creatinine (mg dl^−1^)^a^	253	1.17 (0.93)	1.03 (0.61)	0.13
Platelets (G l^−1^) ^a^	258	163 (92)	192 (102)	< 0.01**
WBC (G l^−1^)^a^	258	10.6 (7.5)	9.1 (5.8)	0.06
Neutrophils (G l^−1^)^a^	224	9 (7)	6.8 (5.6)	< 0.01**

*Statistical significance at *p* < 0.05

**Statistical significance at *p* < 0.01

^a^Non-normal distributed data including median and interquartile range

^b^Categorical parameters are represented as total *n* and percentage

**Table 2:** Bacterial pathogens found in blood culture. Data presented as absolute numbers (*n*) and relative percentage (%) within groups

	*n* (258)	Percent
Gram negative		
*Escherichia coli*	108	78.8
Klebsiella species	20	14.6
Other gram negative bacteria	6	4.4
*Pseudomonas aeruginosa*	3	2.2
Gram positive		
Coagulase-negative staphylococci	48	39.7
*Staphylococcus aureus*	32	26.4
Streptococci	23	19.0
Other Gram positive bacteria	10	8.3
Enterococci	8	6.6

**Fig. 1:**
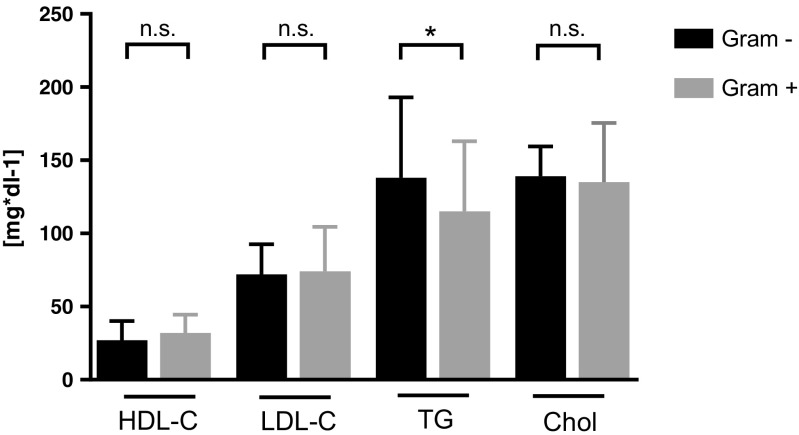
Lipoprotein concentrations in patients with Gram-positive and Gram-negative bacteremia determined within 2 days after hospital admission; groups were compared using Mann-Whitney *U* test. *Significance at *p* value < 0.05. n.s. not significant, HDL-C high-density lipoprotein cholesterol, LDL-C low-density lipoprotein cholesterol, TG triglycerides, Chol total cholesterol

**Fig. 2:**
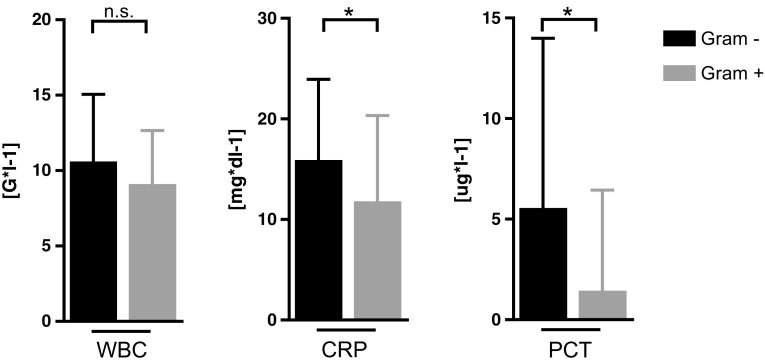
Inflammatory parameters in patients with Gram-negative and Gram-positive bacteremia determined within 2 days after hospital admission; groups were compared using Mann-Whitney *U* test. *Significance at *p* value < 0.05. n.s. not significant, WBC white blood cells, CRP C-reactive protein, PCT procalcitonin

HDL-C, LDL-C, total plasma cholesterol concentrations, and WBC counts did not significantly differ between the two groups at admission (Figs. 1 and 2). However, significantly higher triglyceride (TG) concentrations were found in subjects with Gram-negative than with Gram-positive bacteremia (*p* < 0.05; Fig. 1, Table 1). CRP and PCT concentrations were significantly higher in the Gram-negative group (CRP: *p* < 0.01; PCT: *p* = 0.01; Fig. 2, Table 1).

A linear regression analysis was calculated to predict HDL-C, LDL-C, and TG levels, based on the following independent variables: Gram stain, CRP, WBC, BMI, diabetes, gender, and statin therapy. The generated models for HDL-C and LDL-C were significant (*p* < 0.01, adjusted *r*^2^ = 0.13; *p* < 0.01, corrected *r*^2^ = 0.11), while in the case of TG, it was not significant. CRP (*p* < 0.01) and gender (*p* < 0.01) did significantly impact on HDL-C, while in the LDL-C model, CRP (*p* < 0.01), BMI (*p* < 0.05), and gender (*p* = 0.02) could be identified as variables significantly impacting LDL-C concentrations. Significant correlations between CRP and HDL-C (*r* = − 0.35, *p* < 0.01), LDL-C (*r* = − 0.29, *p* < 0.01), and TG (*r* = 0.18, *p* < 0.01) were found. Similarly, significant correlations were found between PCT and HDL-C (*r* = − 0.39, *p* < 0.01) and LDL-C (*r* = − 0.38, *p* < 0.01), and between WBC and HDL-C (*r* = − 0.13, *p* = 0.04) and LDL-C (*r* = − 0.19, *p* < 0.01), whereas no significant correlations were seen between PCT/WBC and TG.

**Fig. 3:**
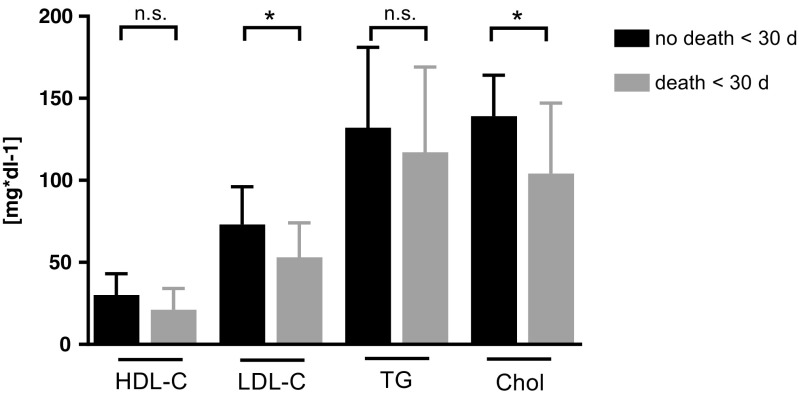
Lipoprotein concentrations of patients who died within < 30 days versus survival > 30 days. Groups were compared using Mann-Whitney *U* test. *Significance at *p* value < 0.05. n.s. not significant, HDL-C high-density lipoprotein cholesterol, LDL-C low-density lipoprotein cholesterol, TG triglycerides, Chol total cholesterol

In terms of prognostic importance, we analyzed correlations between lipoprotein concentrations and length of hospital stay, 30-day mortality, and ICU admission: The median duration of hospitalization was 9 days (Gram-negative = 8 (IQR = 5) vs Gram-positive = 9 (IQR = 10), *p* = 0.3). The duration of hospitalization significantly correlated with CRP (*r* = 0.15, *p* < 0.05) and neutrophil counts (*r* = 0.16, *p* < 0.05), whereas a borderline significance was seen for the correlation between HDL-C levels and length of hospitalization (*r* = 0.12, *p* = 0.06). Patients who died within 30 days after positive blood culture result (*n* = 15) had more often Gram-positive bacteremia (*n* = 11 Gram-positive vs *n* = 4 Gram-negative; *p* = 0.03), significantly lower LDL-C concentrations (median (IQR) 53 mg dl^−1^ (51) vs 73 mg dl^−1^ (47), *p* = 0.01; Fig. 3) and lower total cholesterol concentrations (104 mg dl^−1^ (59) vs 139 mg dl^−1^ (52), *p* < 0.01; Fig. 3) at admission. HDL-C concentrations showed a trend towards lower concentrations in patients who died within 30 days (21 mg dl^−1^ (24) vs 30 mg dl^−1^ (28), *p* = 0.06; Fig. 3). TG, CRP, PCT, white blood cell (WBC) counts, and age were not significantly different between patients who survived and those who died within 30 days (*p* > 0.05).

**Fig. 4:**
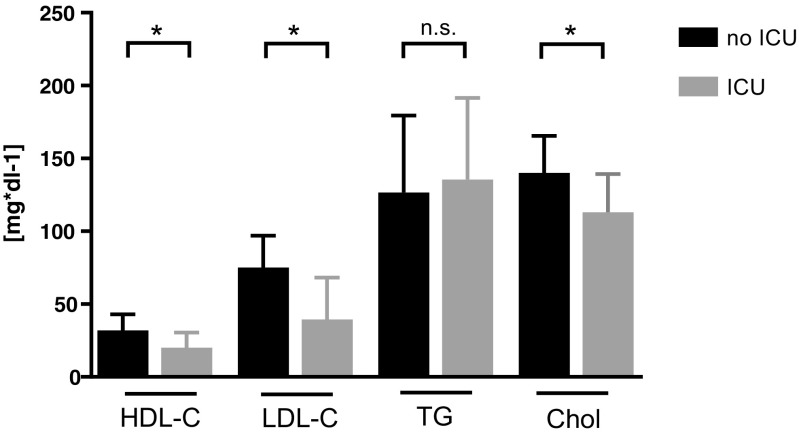
Lipoprotein concentrations of patients requiring ICU treatment. Groups were compared using Mann-Whitney *U* test. *Significance at *p* value < 0.05. ICU intensive care unit, n.s. not significant, HDL-C high-density lipoprotein cholesterol, LDL-C low-density lipoprotein cholesterol, TG triglycerides, Chol total cholesterol

Twenty-six patients were admitted to the ICU (Table 3): These patients were significantly younger (*p* < 0.02), and ICU treatment occurred significantly more frequent in patients with Gram-positive bacteremia (*p* < 0.01). ICU patients had significantly higher CRP (*p* = 0.01) and PCT levels (*p* < 0.01), as well as higher WBC counts (*p* = 0.04). Significantly lower LDL-C (*p* < 0.01), HDL-C (*p* = 0.01), and total cholesterol levels (*p* < 0.01) were found in patients who were admitted to the ICU (Fig. 4). The duration of ICU stay did not significantly differ between the bacteremia groups. No differences in duration of hospital stay, 30 days mortality, or ICU admission were observed between patients with and without statin therapy.

**Table 3:** Prognostic characteristics for ICU admission

		ICU admission	
		*n* (26)	*p*
Gender (m/f)	[n]	17:9	0.22
Gram (neg/pos)	[n]	7:19	< 0.01**
Statin therapy (yes/no)	[n]	9:15	0.29
Mean age ± SD (ICU: : /no ICU)	[y]	62.8 ± 11.5 vs 67.4 ± 18.5	0.02*

*Statistical significance at *p* < 0.05

**Statistical significance at *p* < 0.01

After splitting the HDL-C levels into tertiles (cut-off values at 20 and 37 mg dl^−1^ respectively), significantly more patients died within 30 days and required ICU therapy, if their HDL-C levels were allocated in the lowest tertile (HDL-C range 0–20 mg dl^−1^; *p* = 0.02 and *p* = 0.04, respectively). After splitting the LDL-C levels into tertiles (cut-off values at 55 and 85 mg dl^−1^, respectively), significantly more patients required ICU therapy, if their LDL-C levels were allocated in the lowest tertile (LDL-C range 0–55 mg dl^−1^; *p* < 0.01). No such effects were observed, when splitting TG into tertiles.

Patients with HDL-C concentrations below 20 mg dl^−1^ and LDL-C concentrations below 55 mg dl^−1^ (*n* = 50) had a relative risk (RR) of 2.85 for ICU therapy requirement and a twofold higher risk of death within 30 days.

## Discussion

Early diagnosis and subsequent targeted antibiotic therapy are crucial for the successful treatment of bacteremia. To date, several biomarkers have been studied as diagnostic or prognostic markers of sepsis. Yet, little is known about the potential of dyslipidemia to differentiate for specific bacteria. In our cohort, we were not able to show a clear lipoprotein pattern associated with either Gram-positive or Gram-negative bacteremia. However, the outcome of patients with bacteremia appears to be related to plasma lipoprotein concentrations.

So far, most studies focused on biomarkers potentially related to sepsis, rather than to bacteremia. Most consistent data exist about AP proteins including PCT and CRP, but they have not been analyzed in their ability to determine the underlying microbial pathogen [13]. This study focused on bacteremia in order to compensate the diagnostic uncertainty associated with sepsis, where BC might be negative [4].

Low HDL-C and LDL-C concentrations were observed in both bacteremia groups, as has been extensively described in the literature [8, 14]. Contrary to our expectations, there were no significant differences in HDL-C and LDL-C concentrations between the groups, whereas plasma TG levels, CRP, and PCT were higher in Gram-negative bacteremia patients. We expected to observe a significant difference between Gram-negative and Gram-positive bacteremia, as LPS from Gram-negative pathogens was shown to affect chemokine induction more extensively compared to lipoteichoic acid from Gram-positive bacteria [15, 16]. Even though this hypothesis did not hold true in our analysis, the Gram-negative group differed in the magnitude of inflammatory parameters (CRP, PCT, neutrophils), indicating higher inflammatory activity in the Gram-negative group. This finding is in accordance with an earlier study, suggesting that increased levels of CRP may be suggestive for Gram-negative bacteremia [17]. In contrast to our results, Zou et al. described significant HDL-C and apoA1 depletion in Gram-negative sepsis patients compared to Gram-positive pathogens in a retrospective study (*n* = 76) [18]. According to their longitudinal measurements, they postulated the initial 24-h change in HDL-C as a diagnostic parameter in sepsis patients to differentiate between Gram-negative and Gram-positive pathogens. In comparison to our study, no differences in the magnitude of inflammation indicated as CRP and PCT were seen. Moreover, no information about comorbidities and therapies impacting lipoprotein levels like BMI, diabetes mellitus, parenteral nutrition, or the use of statins were reported, which potentially may have biased the results. No prognostic or outcome-related information were reported.

In our study population, we speculated that higher triglyceride concentrations in Gram-negative bacteremia might be due to the higher inflammatory state as represented by CRP, PCT, and neutrophil counts. Multiple cytokines have been shown to increase triglyceride and triglyceride-rich lipoproteins (VLDL) during an inflammatory state or after LPS challenge [19, 20]. This was shown to be mediated through an increase in de novo lipogenesis as well as a decrease in VLDL clearance. TNF-α, IL-1, and IL-6 mediate the activation of acetyl-CoA carboxylase (ACC), a key regulator of lipogenesis and the suppression of lipoprotein lipase synthesis, hampering efficient triglyceride clearance [21, 22]. However, as measurement and evaluation of TNF-α and IL-1 kinetics were not part of the current study, further investigations are required to elucidate their role in TG synthesis during bacteremia.

Regarding the prognostic relevance of lipoproteins, we were able to show an inverse correlation of HDL-C with the length of hospitalization, as well as lower HDL-C concentrations in patients treated at the ICU. Moreover, patients with HDL-C < 20 mg dl^−1^ had an increased risk of death within 30 days and an elevated frequency of ICU therapy requirement. Reports about HDL-C concentrations and infectious disease risk in humans are limited to a few studies [23–28]. Shor et al. retrospectively reviewed HDL-C levels of 204 hospitalized patients and found that low HDL-C levels were associated with increased odds of fever and sepsis [23]. Especially HDL-C levels < 20 mg dl^−1^ were associated with an increased risk of death, sepsis, and malignancy. Similarly, Rodríguez-Sanz et al. conducted an observational study of 1385 acute ischemic stroke patients and reported an elevated risk for development of infectious complications in patients with HDL-C levels < 38.5 mg dl^−1^ [24]. Grion et al. published a prospective cohort study of 1719 patients, of which 51 developed severe sepsis. In sepsis patients, compared to 71 paired controls, HDL-C levels at admission were identified as a risk factor for severe sepsis [25]. Canturk et al. analyzed HDL-C levels in 418 patients prior surgical procedures and described an association with postoperative nosocomial infections [26]. Chien et al. described low serum HDL-C as a poor prognostic factor of severe sepsis in 63 ICU patients, and low HDL-C correlated with increased 30 days mortality [27]. Highest mortality rates were seen in patients with HDL-C-levels < 20 mg dl^−1^ [27]. No information was given about bacteremia or the spectrum of pathogens. Barlage et al. described a similar situation in 151 patients meeting the sepsis criteria (72% having a positive BC), reporting low HDL-C and LDL-C levels in non-survivors [29]. Interestingly, in their longitudinal measurements, they found an increase in HDL-C levels only in survivors, whereas in non-survivors plasma levels were stable or showed a further decrease until day 11. A recent analysis by Madsen et al. found a u-shaped relationship between HDL-C and risk of infectious disease, especially if HDL-C levels were below 31 mg dl^−1^ or above 100 mg dl^−1^, in a large cohort of 106,553 individuals [28]. In summary, our analysis is in line with all previous studies, supporting the hypothesis that low HDL-C is associated with a worse prognosis also in the setting of bacteremia, independent of sepsis. Especially, HDL-C levels below a range of 20–35 mg dl^−1^ seem to influence the prognosis significantly.

LDL-C concentrations were lower in the non-survivor group, which is in accordance with previous findings suggesting that not only HDL-C but also LDL-C particles may exert a protective function against lethal endotoxemia following Gram-negative infection [30, 31]. However, a clear causality cannot be inferred based on the available data. It might be the case that the severity of the disease led to an LDL-C lowering effect as a consequence of the cytokine storm in the subgroup of patients with a worse outcome and high inflammation [32]. There is evidence, suggesting that proprotein convertase subtilisin/kexin type 9 (PCSK9), a regulator of LDL-C, may also regulate the removal of pathogen lipids such as LPS [33, 34]. Boyd et al. described elevated PCSK9 levels during sepsis as well as a close inverse relation with LPS clearance and a relation with sepsis severity [34]. Walley et al. showed lower inflammatory cytokine responses to LPS challenge in PCSK9 knockout mice compared to wild-type mice and described human PCSK9 loss-of-function genetic variants with improved survival in sepsis, suggesting that lower LDL-C levels improve the overall outcome in Gram-negative infections [33]. However, there is also conflicting evidence, as Guirgis et al. showed that low LDL-C at baseline was associated with higher rates of community-acquired sepsis at a median follow-up time of 3.6 years in a cohort of 29,690 subjects [35]. The study reported several limitations, especially the investigated REGARDS observation cohort that was limited to selected patients, and the initial study was not designed to investigate sepsis. To summarize, we think that the immunologic properties of LDL-C are insufficiently represented by LDL-C mass. The here presented low LDL-C levels are probably the result of a high inflammatory state, which is also supported by the highly significant correlation between LDL-C levels and the magnitude of inflammation represented by CRP, PCT, and WBC.

Interestingly, we did not observe an association between high levels of TG and outcome parameters, although high TG is often present in individuals with poor health status, and therefore one could hypothesize an impact on outcome parameters.

The strengths of this study are its double-center design, the cohort size, and homogeneity regarding confounders influencing lipid levels, especially parenteral nutrition, malignancy-associated dyslipidemia, BMI, diabetes mellitus, and the use of statin therapy. Major limitations of this study are its retrospective design and the single blood sample at admission, although plasma lipoprotein levels are fairly stable in the initial assessment and have adequate intraindividual reproducibility. Moreover, the present study only investigated lipoproteins and inflammatory parameters (CRP and WBC) in their ability to differentiate between Gram-positive and Gram-negative bacteremia and their potential as prognostic markers at the onset of disease.

Future studies addressing the cholesterol metabolome in bacteremia and sepsis should especially focus on follow-up data during the initial sepsis phase and measure lipoprotein function, in order to differentiate between cholesterol mass and function in terms of immunological properties, LPS clearance, and (reverse) cholesterol transport.

## Conclusion

The practical implication of the here presented study is the prognostic relevance of lipoprotein concentrations in patients with bacteremia. The outcome seems closely linked to lipoprotein concentrations, but further studies are required until measurement of lipoproteins in bacteremia may be recommended to help in early identification of subjects at increased risk of a worse outcome. A differentiation between Gram-positive and Gram-negative pathogens by using lipoprotein patterns is unlikely.

## Data Availability

The datasets generated during and/or analyzed during the current study are available from the corresponding author on reasonable request.
